# Editorial: Machine Learning With Radiation Oncology Big Data

**DOI:** 10.3389/fonc.2018.00416

**Published:** 2018-09-27

**Authors:** Jun Deng, Issam El Naqa, Lei Xing

**Affiliations:** ^1^Department of Therapeutic Radiology, Yale University, New Haven, CT, United States; ^2^Department of Radiation Oncology, University of Michigan, Ann Arbor, MI, United States; ^3^Department of Radiation Oncology, Stanford University, Stanford, CA, United States

**Keywords:** big data, machine learning, artificial intelligence, personalized medicine, personalized radiotherapy

## Introduction

Half of all cancer patients may receive radiotherapy as part of their treatment. With the wealth of diverse data generated every day in the clinic, the radiation oncology community possesses a unique advantage in harnessing these massive data with the predictive power of machine learning methods for the benefit of millions of cancer patients undergoing radiotherapy worldwide. In this Research Topic “Machine Learning with Radiation Oncology Big Data,” a wide range of clinical applications involving various machine learning algorithms have been described and demonstrated, with the hope of ushering in more widespread applications of artificial intelligence in medicine, particularly in cancer radiotherapy in order to achieve a truly individualized radiation oncology and an evidence-based learning healthcare system.

## Topics covered in this research topic

- Knowledge-based treatment planning: Zhang et al.
- Knowledge-based response-adapted radiotherapy: Tseng et al.
- Radiomics image analysis: Elhalawani et al., Jethanandani et al.
- Radiogenomics and outcome modeling: Kang et al.
- Automated contouring and nodule detection: Jackson et al.,  Ali et al., Men et al.
- Comparative effectiveness research: Sanders and Showalter.
- Machine learning in radiation oncology overview: Feng et al.
- Normal tissue complication probability modeling: Gabryś et al.

## Papers included in this research topic

In this review paper, Elhalawani et al. summarized the feedback of eight contestants who participated in a recent radiomics challenge in head and neck radiation oncology, and discussed some of the challenges in sharing and directing existing datasets toward clinical implementation of radiomics in radiation oncology.

Tseng et al. discussed recent development in the knowledge-based response-adapted radiotherapy for personalized radiotherapy management. They addressed three specific questions that are necessary to realize it clinically: (1) what knowledge is needed, (2) how to estimate radiotherapy outcomes accurately, and (3) how to adapt optimally.

Kang et al. presented an overview of machine learning algorithms in the application of radiogenomics to combine genomics signatures with radiotherapy. They summarized the important lessons learned for the proper integration of machine learning into radiogenomics analysis.

Jackson et al. introduced a convolutional neural network approach for fully automated contouring of kidneys and automated radiation dose estimation in an unsealed source therapy, which provides comparable accuracy to humans while largely reducing the planning time.

In a systematic review, Jethanandani et al. explored the various applications of radiomics in magnetic resonance imaging of head and neck cancer, and identified the lack of standardization in study design as a major limitation to their clinical relevance.

Sanders and Showalter described their vision of combining big data with comparative effectiveness research methodologies within the framework of a rapid-learning healthcare system in order to accelerate discovery and realize a fully individualized radiation treatment.

Feng et al. identified specific opportunities in a long chain of radiotherapy processes where machine learning could improve the quality and efficiency of patient care in radiation oncology, as well as the needs required to realize them at both the community and institutional levels.

Ali et al. presented a robust non-invasive deep reinforcement learning method to predict the presence of lung nodules, a common precursor to lung cancer, based on 888 lung CT scans of the lung nodule analysis (LUNA) challenge.

Zhang et al. proposed an ensemble approach to knowledge-based intensity modulated radiation therapy treatment planning, and demonstrated its advantages in terms of robustness against small training set sizes, mis-labeled cases, and dosimetric inferior plans.

Gabryś et al. investigated whether machine learning with dosimetric, radiomic, and demographic features can allow for more precise xerostomia risk assessment. They identified the need for the development of personalized data-driven risk profiles for normal tissue complication probability (NTCP) modeling.

Men et al. developed an end-to-end deep deconvolutional neural network for segmentation of nasopharyngeal tumor volumes to improve the consistency of contouring and streamline radiotherapy workflows, but cautioned that careful human review and a considerable amount of editing would still be required.

## Conclusions and outlook

The 11 papers included in this Research Topic produced some promising results and offered visionary perspectives regarding the role of machine learning with radiation oncology big data. The clinical applications demonstrated here are considered just the tip of the iceberg of the incoming full-spectrum applications of human intelligence and artificial intelligence in radiation oncology. While still in its infancy stage, we envisage that artificial intelligence together with human intelligence can provide something much better than either one could perform alone in the near future.

## Author contributions

All authors listed have made a substantial, direct and intellectual contribution to the work, and approved it for publication.

### Conflict of interest statement

The authors declare that the research was conducted in the absence of any commercial or financial relationships that could be construed as a potential conflict of interest.

